# Moving beyond antenatal care: Need for a life-course approach to women’s health, South Africa

**DOI:** 10.4102/safp.v67i1.6202

**Published:** 2025-11-14

**Authors:** Jagidesa Moodley, Yogan Pillay, Olive P. Khaliq

**Affiliations:** 1 Department of Obstetrics and Gynaecology, College of Health Sciences, University of KwaZulu-Natal, Durban, South Africa; 2 Department of Public Health, Faculty of Health Sciences, University of Pretoria, Pretoria, South Africa; 3 Department of Paediatrics and Child Health, Faculty of Health Sciences, University of the Free State, Bloemfontein, South Africa

## Introduction

South Africa has made significant progress in improving maternal and perinatal health in recent decades. The introduction of universal access to antenatal care with the 8-visit schedule improved human immunodeficiency virus (HIV) clinical management and expansion of emergency obstetric services, contributing to significant reductions in maternal and neonatal morbidity and mortality. However, challenges still exist, as rates of preterm births, hypertensive disorders of pregnancy and stillbirths remain high, particularly among disadvantaged communities.^1^

One of the reasons for the need for greater progress is that most interventions continue to focus narrowly on the pregnancy period itself. By the time a woman presents for her first antenatal visit, many of the risks for adverse outcomes have already been established, often years before conception. To decrease negative outcomes further, the need to move beyond episodic pregnancy care and adopt a life-course approach to women’s health, with an emphasis on preconception and interconception care, is required.

## Why is a life-course approach required?

South Africa, like many low- and middle-income countries (LMICs), is now facing a double burden of disease, rising rates of non-communicable diseases alongside ongoing infectious disease challenges. Increasing numbers of women of reproductive age are entering pregnancy with obesity, diabetes, hypertension and HIV – conditions that significantly raise the risk of maternal and neonatal complications.^2^ Moreover, many women experience short inter-pregnancy intervals, unresolved complications from previous pregnancies and undiagnosed mental health issues.^3^ In addition, the postpartum period is often a neglected window for continuing healthcare, with limited follow-up or structured support. As a result, opportunities to promote recovery and reduce the risk of recurrence of medical conditions are missed.

*Preconception care and interconception care* provide a framework to address these gaps. Preconception care includes health promotion, screening for chronic diseases and infections, nutritional interventions and reproductive life planning provided prior to pregnancy.^4^ Interconception care focuses on improving health between pregnancies, with emphasis on birth spacing, mental health and managing prior medical and socio-economic issues.^5^

Both forms of care (preconception and interconception care) share a common goal: to prepare women for healthy pregnancies and to improve their long-term health.

### Potential impact in the South African context

Adopting a life-course approach could have a particularly powerful impact in South Africa. For example:

- Optimising glycaemic control *before conception* among women with diabetes could prevent congenital anomalies, miscarriage and stillbirths.^6^
- Ensuring adequate *folic acid supplementation* could reduce the incidence of neural tube defects.
- Identifying and treating *hypertension and obesity* before pregnancy could lower rates of pre-eclampsia, placental abruption and preterm birth.
- Promoting HIV awareness and regular testing in communities.
- Ensuring that all teenagers receive Human Papillomavirus (HPV) vaccinations.
- Addressing *mental health* and *gender-based violence* in the postpartum period could improve maternal well-being and reduce adverse outcomes in subsequent pregnancies.
- Promoting healthy *birth spacing* could reduce risks of pregnancy complications such as preterm birth and low birth weight babies.^7^
- Promoting healthy lifestyles, including appropriate and affordable diets and regular physical exercises or activities.
- Ensuring that learning institutions (schools and higher education) introduce and promote social and reproductive health issues for all scholars to overcome the issues of teenage pregnancies and sexually transmitted infections.

Importantly, these interventions are not high-cost or technology-dependent – they are achievable through better integration of services within the existing primary health system and the community health worker programmes.

## Barriers to implementation

Despite the clear benefits, preconception and interconception care remain underdeveloped in South Africa. Several barriers persist:

- *Fragmented care*: Family planning, HIV services, Noncommunicable diseases (NCD) management and maternity care are delivered in silos, with little continuity.
- *Limited awareness*: Many women and healthcare providers do not recognise the importance of preconception health.
- *Resource constraints*: Overburdened primary care facilities may lack capacity for systematic preconception interventions.
- *Social barriers*: Stigma around contraception and limited reproductive autonomy for many women.
- *Partner healthcare*: Many men, including adolescents, do not visit clinics for healthcare and pose a risk for sexually transmitted infections.

## Opportunities for action

There is an urgent need for **policy and programmatic action** to strengthen preconception and interconception care in South Africa:

- *Integrate preconception care* into routine services at the primary care level, including NCD clinics, HIV services and family planning.
- Use all health system contacts – for contraception, child immunisation, or chronic disease care – as opportunities to address women’s reproductive health goals.
- *Train healthcare workers, including community health workers* in life-course approaches, enabling them to identify risks and provide counselling before and between pregnancies for women and their partners.
- *Strengthen postpartum care*, including screening for chronic diseases, mental health and reproductive planning.
- Develop *public awareness campaigns* to empower women to seek preconception care.
- Include indicators of preconception and interconception care in national maternal health monitoring frameworks.
- Include preconception and interconception care services in men who want to conceive.

In the Netherlands, Healthy Pregnancy 4 All has been established to improve pregnancy outcomes. There are two obstetric interventions, one before pregnancy (programmatic preconception) and one during pregnancy (systematic antenatal risk assessment).^8^ This intervention would be suitable to try in a South African setting, as this study used it in geographical areas with high adverse perinatal outcomes. During the intervention, community-based primary providers, such as general practitioners and midwives, provide preconception care consultations. There are two sessions in these consultations. The woman completes a questionnaire before the first session. This questionnaire examines risk variables in the following areas: family, work and/or environment, medical history, obstetric and/or gynaecologic history, lifestyle and background. A history of possible risk factors is obtained during the consultation, and the pregnant woman develops an intervention strategy to lessen or eliminate risk factors. A follow-up visit is scheduled for 3 months later to assess compliance with the intervention plan.

The World Health Organization (WHO) created a life-course approach to sexual and reproductive health. This is guidance on preconception and interconception care as a core life course for sexual reproductive health (SRH) and mother, newborn, child and adolescent health (MNCAH).^9^ In South Africa, a practical implementation blueprint can be used:

### Policy and governance

Adopt a national Life-Course Policy Note (annex to the *Maternal, Newborn, and Child Health & Prevention of Mother-to-Child Transmission [PMTCT] policy)* that

- Defines service packages by life stage (adolescence, preconception, pregnancy, postpartum and/or interconception, early childhood).
- Mandates such as joint Reproductive, Maternal, Newborn, Child, Adolescent Health and Nutrition (RMNCAH+N) planning across Primary Healthcare (PHC), HIV, NCDs, sexual reproductive health, mental health and social services.

### Service packages by life stage

#### What to deliver

- *Adolescents (school and youth-friendly services)*: GBV assistance, SRH awareness, HPV vaccination, contraception, HIV testing and pre-exposure prophylaxis (PrEP) where necessary, and nutrition and mental health screening.
- *Preconception (women and men)*: Tuberculosis (TB) symptom screening where appropriate, folate and/or anaemia and/or nutrition, contraception for birth spacing, targeted non-communicable disease optimisation, fertility counselling and risk screening (HIV, syphilis, diabetes and hypertension).
- *Pregnancy*: Maternal immunisation, TB/HIV/NCD integration and routine antenatal care with risk-stratified routes (medical and societal risks).
- *Postpartum and interconception (up to 12 months)*: Organised postnatal check-ups (6 weeks, 3 months, 6 months, and 12 months) for mental health, cardiovascular diseases (CVD) risk, diabetes/hypertension management, contraception, tuberculosis prevention treatment (TPT) as necessary and return to chronic care for long-term problems.
- *Early childhood (0-5-years-old)*: Nutrition, immunisations, growth and neurodevelopment monitoring, and early learning referrals – all utilising the ChCC-style ‘every contact counts’ methodology. These priorities are in line with the South African perspective that was uploaded regarding preconception and/or interconception care and its anticipated effects on congenital abnormalities, hypertensive disorders, stillbirths and preterm deliveries.

### Data and digital tools

#### Build once, use everywhere

- *Person ID spine*: Invest in probabilistic matching to eliminate past duplication and set the Health Patient Registration System as the default ID for primary healthcare, maternity, HIV/TB/NCD, private, pharmacy and employer systems.
- *Interoperability*: Connect TIER.Net, MomConnect, DHIS2, HPRS, eLabs, private laboratory, and hospital electronic medical records (EMRs) via open standards (Health Level Seven International Fast Healthcare Interoperability Resources [HL7 FHIR], District Health Information Software 2 [DHIS2] Application Programming Interfaces [APIs]).
- *Life-course registry logic*: Set up event-based triggers. For instance, an antenatal care appointment generates a list of postpartum and interconception tasks, and a postpartum visit initiates preconception counselling for the following 6–12 months.

### Artificial intelligence where it adds value

- Risk prediction for example preeclampsia, gestational diabetes mellitus (GDM), postpartum depression, default risk.
- Eligibility nudges (folate, TPT, contraception).
- Linkage automation (eLabs positive → same-day messaging to patient and/or community health worker and referral).
- *Dashboards*: One Reproductive, Maternal, Newborn, Child, Adolescent Health and Nutrition (RMNCAH-N) life-course dashboard consolidating coverage, quality and equity (by district, age, parity and adolescent status).

## Conclusion

To further reduce maternal and neonatal mortality, South Africa must move beyond a narrow focus on antenatal care and embrace a *life-course approach* that addresses women’s health before, between and after pregnancies. Preconception and interconception care for both women and men should be recognised as essential components of comprehensive maternal and neonatal health services; integration of these components into primary care and community engagements are the next critical steps in improving the long-term well-being for women and their families.
